# A Systematic Review: The Impact of COVID-19 Policy Flexibilities on SNAP and WIC Programmatic Outcomes

**DOI:** 10.1016/j.advnut.2024.100361

**Published:** 2024-12-20

**Authors:** Mayra Crespo-Bellido, Gabby Headrick, Miguel Ángel López, Jennifer Holcomb, Ariana Khan, Shanti Sapkota, Kelseanna Hollis-Hansen

**Affiliations:** 1 Center for Nutrition and Health Impact, Omaha, NE, United States; 2 Department of Exercise and Nutrition Sciences, Milken Institute of Public Health, George Washington University, Washington, DC, United States; 3 Sinai Chicago, Sinai Urban Health Institute, Chicago, IL, United States; 4 Department of Public Health, University of Texas Southwestern Medical Center, Dallas, TX, United States

**Keywords:** SNAP, WIC, program access, enrollment, retention, benefit utilization, policy flexibilities

## Abstract

In response to the coronavirus disease 2019 (COVID-19) public health emergency (PHE), the federal government deployed policy flexibilities in food and nutrition assistance programs including the Supplemental Nutrition Assistance Program (SNAP) and Special Supplemental Nutrition Program for Women, Infants, and Children (WIC) to meet the needs those experiencing economic hardship. Emergent literature evaluates the impact of these flexibilities on program outcomes. The objective of this study was to explore the impact of policy flexibilities deployed during the COVID-19 PHE on access, enrollment/retention, benefit utilization, and perceptions of SNAP and WIC. Keyword searches were performed in November 2023, February 2024, and August 2024. The search included peer-reviewed literature from 2020 to 2024, following Johanna Briggs Institute (JBI) and Preferred Reporting Items for Systematic Reviews and Meta-Analyses guidelines, resulting in 37 eligible articles. Twelve studies evaluated policy flexibilities in SNAP only, 21 in WIC only, and 4 in both programs. Across these, 12 studies explored program access, 7 enrollment/retention, 13 benefit utilization, and 15 program perceptions. JBI critical appraisal tools were used to assess risk of bias. The reviewed articles show that although SNAP and WIC participants identified challenges to access, there were increases in enrollment/retention due to policy flexibilities enabling remote services and reducing administrative burden in both programs. Benefit increases led to greater purchase of preferred foods in SNAP and greater access to fruit and vegetables in WIC. Overall, participants were satisfied with the flexibilities and reported most were beneficial for their households. Some implementation challenges were identified by participants and staff. A few studies showed potential risks of bias, including selection bias and confounding bias. COVID-19-related policy flexibilities in SNAP and WIC demonstrated significant improvements in selected program outcomes; however, challenges communicating policy flexibilities to authorized vendors and participants created difficulties to benefiting from the flexibilities. Findings from the evaluations of these flexibilities can inform future program enhancements and long-term regulatory changes. This study was registered in PROSPERO (CRD42023493302).

**Statement of Significance**

The exploration of how Supplemental Nutrition Assistance Program and Women, Infants, and Children policy flexibilities supported program access, enrollment, and retention provide valuable insights that may help guide equitable improvements in service delivery as the federal government prioritizes modernization efforts.

## Introduction

The USDA administers 16 food and nutrition assistance programs designed to mitigate food insecurity and provide low-income households with access to nutritious foods. Two of the largest programs serving eligible United States households are the Supplemental Nutrition Assistance Program (SNAP) and the Special Supplemental Nutrition Program for Women, Infants, and Children (WIC). In 2022, SNAP served over 41 million people and WIC reached 6.3 million [1,2]. Although these means-based programs have distinctions from one another, they share many commonalities. Applicants must be deemed eligible through an interview (SNAP) or a nutrition risk assessment (WIC) [3,4]. Upon approval, people receive an Electronic Benefit Transfer (EBT) card to acquire eligible food items at authorized retailers. SNAP allows most foods, except hot-prepared foods and alcohol, whereas WIC prescribes food packages tailored to promote health for pregnant, breastfeeding, and postpartum women, infants, and children [3,4].

Research consistently shows that these programs improve household food security. SNAP participation is estimated to reduce food insecurity by ∼30% after accounting for the likelihood that households experiencing the worst food hardships are more likely to apply [5,6]. WIC promotes food security among participants, with food insecurity risk increasing once participation stops [7]. Children receiving both SNAP and WIC have higher food security odds through the life course compared with income-eligible children participating in only 1 program or none [8].

Despite improvements in economic and food security for those receiving SNAP and WIC, many households remain food insecure due to inadequate benefit amounts across programs [9,10]. Many families report that SNAP benefits are exhausted within 1–2 wk of issuance, making it challenging to meet food needs toward the end of the benefit month [11,12]. Furthermore, although WIC participation is associated with healthier food purchasing and diet, families often found it difficult to afford fresh fruits and vegetables due to insufficient benefit allocation for these items within pre-pandemic prescribed food packages [10]. Research examining the impact of increased benefit allocations for SNAP and WIC has found improved food security for participating households, demonstrating a policy pathway to address shortcomings [13,14].

Beyond benefit inadequacy, barriers related to eligibility, enrollment, and retention create significant challenges for households with lower income [15]. On average, ∼84% of eligible individuals participated in SNAP in 2017; however, participation varies by state from as low as 50% of eligible people receiving benefits (Wyoming) to nearly 100% of eligible people receiving benefits (Oregon, New Mexico, Vermont, Rhode Island, Delaware, Illinois) [16]. For WIC, participation rates have remained between 50% and 55% over the past 5 y [17]. Processes required to establish and maintain participation, including paperwork delays, difficulties accessing application and recertification portals, and in-person interview requirements, are cited as substantial administrative barriers for those trying to access or retain benefits [12,18].

In response to COVID-19 pandemic economic and social hardships, Congress and USDA approved several program flexibilities (that is, waivers) through the Families First Coronavirus Response Act in March 2020. These flexibilities aimed to promote equitable and simplified access to SNAP and WIC during a time of great need [19]. The flexibilities suspended certain program requirements, such as work mandates and in-person nutrition clinic visits, increased benefit amounts, expanded access to online grocery purchasing, and simplified application and recertification procedures [20]. Many of these temporary measures were extended through a Continuing Resolution enacted in October 2020, allowing the continued implementation of the waivers into 2021 and 2022 [20].

Since implementation of temporary program flexibilities, many studies have explored their impact on program access, enrollment, retention, and benefit utilization outcomes. This systematic review examined the association of COVID-19-related SNAP and WIC policy flexibilities with each of these program-related outcomes, focusing on households with children and programs’ staff.

## Methods

Following the PRISMA guidelines and Johanna Briggs Institute (JBI) recommendations for critical appraisal [21], we systematically reviewed the effect of pandemic SNAP and WIC policy flexibilities on program access, enrollment, retention, benefit utilization, and program perceptions. Our protocol was prospectively registered in PROSPERO and is available at: https://www.crd.york.ac.uk/prospero/display_record.php?ID=CRD42023483486.

### Exposure of interest

We included articles published between March 2020 and August 2024 focusing on 17 SNAP and/or WIC COVID-19-related policy flexibilities. Table 1 names and defines all policy flexibilities identified.

**TABLE 1:** Supplemental Nutrition Assistance Program (SNAP) and Special Supplemental Nutrition Program for Women, Infants, and Children (WIC) COVID-19 policy flexibilities evaluated in a systematic review on programmatic outcomes.

Policy evaluated	Abbreviation	Definition	Earliest date allowed	Date lapsed for all states^1^
SNAP Adapted Telephonic Signature Requirements Waiver	TSRW	USDA allowed states to document the household's verbal attestation to the information on the application, instead of requiring an audio recording to constitute a valid telephonic signature.	March 2020	September 2022
SNAP Emergency Allotments	EAP	USDA granted waivers to states to allow issuance of emergency allotments (supplements) for SNAP households as long as federal government declares public health emergency and states issued an emergency/disaster declaration.	March 2020	February 2023
SNAP Expedited Interview Waiver	EIW	FNS allowed states to not require interviews before approval for households eligible for expedited service after identification and attempt made to contact the household for an interview.	March 2020	September 2022
SNAP Extended Certification Periods and adjust Periodic Reports Waiver	ECRW	FNS allowed states to extend certification periods and temporarily adjust periodic report form submissions due for SNAP household.	March 2020	September 2022
SNAP Interview Waiver for Initial/Recertification Interviews	IRW	FNS allowed states to not require a household to do an interview before approval after identity and other mandatory verification.	March 2020	September 2022
SNAP Interview Waiver to not offer Face-to-Face Interviews	NFIW	FNS allowed states to not offer face-to-face interview or granting a request for face-to-face interview to any household at application/recertification.	March 2020	September 2022
SNAP Periodic Reporting Procedures Waiver to Recertify Households	PRRW	This waiver allowed states to extend flexibility for SNAP households using periodic reporting rather than extensive recertification process.	April 2020	November 2022
SNAP Thrifty Food Plan Universal Benefit Increase	TFPI	The Thrifty Food Plan serves as the basis for setting maximum SNAP benefits in every federal fiscal year. Due to revision of Thrifty Food Plan, the benefits were raised for SNAP households.	October 2021	Ongoing
SNAP waiver to conduct quality control interviews in person	QCIP	FNS allowed temporary waivers to conduct telephone quality control interviews, instead of face-to-face.	June 2020	September 2024
WIC Cash-Value Voucher/Benefit (CVV/B for fruits and vegetables)	CVVI	Under the ARPA, the USDA temporarily increased the CVV/B to $35/mo per person, initially for 4 mo. In October 2021, the CVV/B was adjusted to $24/mo and to $25/mo in October 2022 ($25 for child participants, $44 for pregnant and postpartum participants, and $49 for fully and partially breastfeeding participants).	June 2021	Ongoing
WIC Extended Issuance (4 mo at a time vs. 3 mo at a time)	EI	The FFCRA gave FNS authority to provide an extension of the certification period of ≤90 d for a child receiving Food Package IV category only.	March 2020	May 2023
WIC Food package substitutions	FPS	Waivers allowed approved State Agencies and Tribal Agencies to expand the list of approved foods by permitting appropriate substitutes for the types and amounts of certain WIC-prescribed foods if their availability is limited or not available. For example, WIC families could substitute milk of any available fat content if prescribed varieties were not available.	March 2020	May 2023
WIC Physical Presence Waiver	PPW	The waiver allowed all individuals to enroll or re-enroll without visiting a clinic in-person and postpone medical tests for states that make it a requirement. For example, includes waiver to defer certain physical features and bloodwork requirements to evaluate nutrition risk.	April 2020	May 2023
WIC Remote Benefit Issuance Waiver	RBI	The FFCRA gave FNS authority to remove barriers for remote issuance of WIC benefits to minimize potential exposure. For example, participants would not have to come into clinic to pick up EBT cards or paper coupons.	March 2020	May 2023
WIC Separation of Duties Waiver	SDW	The waiver allowed single staff to determine eligibility for all certification criteria and issuing food instruments, cash-value vouchers, or supplemental food for same participant at WIC agencies to promote social distancing at the time of certification.	March 2020	May 2023
WIC Transaction without Presences of a Cashier	TPC	The waiver allowed participants to make WIC purchases without the presence of a cashier. For example, online ordering and curbside pickup on WIC-authorized retailers.	September 2020	May 2023
WIC Vendor Preauthorization Flexibilities	VPF	The waiver removed the federal requirement that State Agencies must conduct an onsite visit before or at the time of a vendor’s initial authorization.	May 2020	May 2023

1 There was high variability between states when each of the policies lapsed.

### Outcomes of interest

Outcomes included program access, enrollment, retention, benefit utilization, and perceptions on policy flexibilities. We define access as the ability of households to enroll in and receive benefits without encountering significant barriers. Enrollment was defined as new participants brought on and retention as participants re-enrolling or recertification enabled by flexibilities. Benefit utilization was defined as food purchasing patterns using EBT or total benefit dollars spent. Participant perceptions on flexibilities were defined as participant satisfaction, experiences, and reasons for remaining in the program.

### Population of interest

SNAP and/or WIC participants or caregivers, or SNAP and/or WIC program administrators.

### Studies of interest

We included quantitative (e.g., cross-sectional surveys, natural experiments), qualitative (e.g., focus groups, in-depth interviews), and mixed-methods designs. Our review included articles published in English and geographic areas where SNAP and WIC are implemented (that is, United States and its territories). We included articles from peer-reviewed journals, doctoral-level dissertations, and policy evaluations from nonacademic/gray literature. We excluded systematic reviews, narrative reviews, randomized controlled trials, commentaries, and master-level theses.

### Search strategy

We conducted systematic searches with structured search strings for articles focused on SNAP and WIC COVID-19 policy flexibilities, respectively. Example search strings are in Supplemental Table 1. We conducted searches using SCOPUS, USDA National Agricultural Library, ProQuest, and PubMed. We identified a list of specific organizations known for conducting evaluations for federal entities (e.g., Mathematica, Westat, Abt Associates, Insight Policy Research, National WIC Association, Research and Development, Research Triangle Institute, American Public Human Services Association, Urban Institute, Center for Budget and Policies Priorities, Food Research & Action Center) and conducted searches to identify nonacademic/gray literature published. Gray literature that met the inclusion criteria for exposure, outcomes, population and study designs of interest were included for screening. Among identified articles, we hand-searched reference lists to ensure completion of our included article list. We conducted this systematic search 29 November 2023, 21 February 2024, and 21 August 2024, to assure recent literature was captured.

### Study selection

**FIGURE 1:**
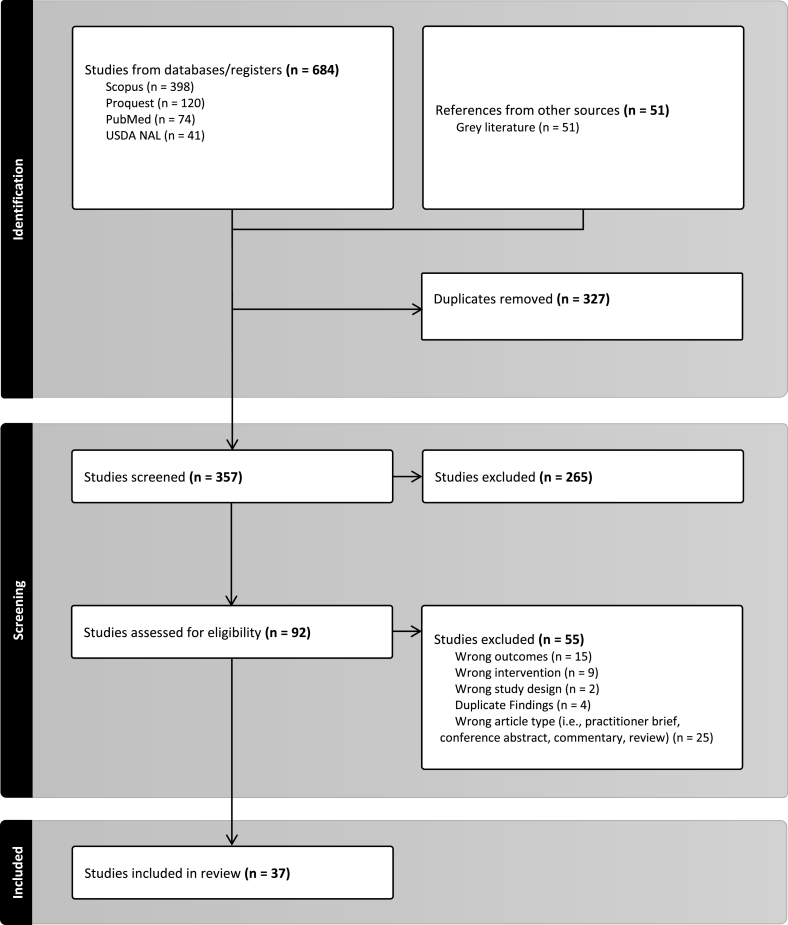
PRISMA flow diagram for a systematic review examining how COVID-19-related policy flexibilities on the Supplemental Nutrition Assistance Program (SNAP) and the Special Supplemental Nutrition Program for Women, Infants and Children (WIC) impacted program access, enrolment, retention, benefits utilization, and program perspectives.

Identified articles were imported into Covidence, a systematic review data management software. Two research assistants double-screened all titles and abstracts against predefined inclusion criteria. Discrepancies were resolved by a third content-expert of the study team. After title and abstract screening, full-texts were double-screened for inclusion by research assistants. In meetings, discrepancies in agreement were resolved through discussion and content-expert review to arrive at our final sample (Figure 1).

### Data extraction

A data extraction form was developed in Covidence and piloted on a quantitative and a qualitative study by 5 team members, who then revised the extraction form for use. Included articles were divided among all team members for extraction, with one team member assigned the role of primary extractor and another assigned as data checker for a total of 2 team members assigned to each article in order to promote data quality and consistency across studies extracted. Fields extracted included study design, aims, sample, methodology, geography, policy flexibility exposures of interest, and outcomes reported.

### Data synthesis

We used a narrative and descriptive synthesis approach to summarize the data, given the diversity and heterogeneity of data types, exposures, and outcomes. Findings were organized by program focus (SNAP, WIC), methodology, data source, study population, and the policy flexibilities examined as exposures. We summarized findings by program focus (SNAP, WIC, or both), methodology, data source, study population, policy flexibilities examined as exposures (Table 2) and outcomes of interest: *1*) program access; *2*) enrollment, retention; *3*) benefit utilization; and *4*) policy flexibility perceptions.

**TABLE 2:** Characteristics of studies included in a systematic review examining how COVID-19-related policy flexibilities to the Supplemental Nutrition Assistance Program (SNAP) and the Special Supplemental Nutrition Program for Women, Infants, and Children (WIC) impacted program access, enrollment/retention, benefits utilization, and program perceptions.

	Overall^1^ (*n* = 37)	SNAP^2^ (*n* = 16)	WIC^3^ (*n* = 24)
Sample sizes, mean (range)	1,163,481 (4–31,884,659)	2,899,239 (22–31,884,659)	281,040 (4–5,294,466)
Investigation type, *n* (%)			
Qualitative	18 (48.6%)	8 (50.0%)	12 (50.0%)
Quantitative	16 (43.2%)	7 (43.8%)	10 (41.6%)
Mixed methods	3 (8.1%)	1 (6.2%)	2 (8.3%)
Data source, *n* (%)			
Administrative data	9 (24.3%)	5 (31.3%)	5 (20.8%)
Surveys	11 (29.7%)	3 (18.8%)	8 (33.3%)
Open-ended survey answers	3 (8.1%)	3 (18.8%)	1 (4.1%)
Interviews	15 (40.5%)	4 (25.0%)	12 (50.0%)
Focus groups	5 (13.5%)	3 (18.8%)	2 (8.3%)
Respondents, *n* (%)			
Participants	33 (86.4%)	13 (81.3%)	24 (95.5%)
Staff	6 (16.2%)	3 (18.8%)	3 (13.6%)
Outcomes of interest, *n* (%)			
Access	12 (32.4%)	5 (31.3%)	8 (33.3%)
Enrollment/retention	7 (18.9%)	4 (25.0%)	4 (16.7%)
Benefit utilization	13 (35.1%)	4 (25.0%)	9 (37.5%)
Program perceptions	15 (40.5%)	4 (25.0%)	12 (50.0%)

Several studies included multiple outcomes, data sources, and populations of interest.

1 Percentages are calculated with the total number of included articles as denominator.

2 Percentages are calculated with the number of articles that included SNAP outcomes as denominator.

3 Percentages are calculated with the number of articles that included WIC outcomes as the denominator.

For quantitative findings, we presented effect measures provided by the authors: proportions/percentages, odds ratios, and mean differences. For qualitative findings, a vote counting method (that is, approach to summarizing evidence by counting studies based on the direction of the results) was employed to assess whether the studies overall found a positive, neutral, or negative effect of the policy changes on SNAP and WIC program outcomes.

Separate tables for SNAP and WIC presented study identifiers, quality assessment, policy change details (that is, which policies were implemented in each state), operationalization of the variables, and relevant findings. These tables aligned with the narrative synthesis discussing access/outreach, enrollment, retention, perception/satisfaction, and benefit use. Because of the heterogeneity of outcome measures, conducting a meta-analysis was not feasible. A narrative and descriptive synthesis was used given the diversity and heterogeneity of data type, exposures, and outcomes.

### Quality appraisal

We evaluated study quality using the JBI critical appraisal tools to assess trustworthiness, relevance, potential sources of bias, and results of included studies [21]. Two team members appraised each article. The JBI critical appraisal tool that best aligned with the study methodology was used to appraise each study. To explore risk of bias, cross-sectional studies were evaluated for inclusion criteria, subject and setting descriptions, exposure measurement validity, and statistical appropriateness. Quasi-experimental studies were assessed for pre- and post-intervention measurements, follow-up completeness, consistency in outcome measurement, and data reliability. Qualitative studies were reviewed for methodological congruity, reflexivity, ethical approval, and representation of participant voices. For mixed-methods studies, the JBI critical appraisal tools that best aligned with both quantitative and qualitative method(s) utilized were used for appraisal. Disagreements were resolved in full team discussions.

A critical appraisal score was assigned to each study. Critical appraisal scores were calculated as the proportion of criteria from the JBI critical appraisal tools that were present in the study, out of all the applicable critical appraisal tool criteria. Critical appraisal scores were interpreted for methodological quality as: <0.39 very weak, 0.40–0.59 weak, 0.60–0.79 moderate, 0.80–0.90 strong, >0.90 very strong.

## Results

We identified 633 studies from PubMed, Scopus, ProQuest, and Agricola, and 51 studies from other sources (that is, reports from professional organizations, nonprofit evaluations). Of these studies, 326 were identified as duplicates and excluded. The remaining 332 studies underwent screening; in which 248 were excluded after title and abstract/summary screening for not aligning with the review’s objective. An additional 51 studies were excluded during full-text review for not meeting eligibility criteria. Thirty-seven studies were included in the final analysis. Figure 1 includes an illustration of the search, selection, and screening process.

### Study characteristics

Of 37 studies that met inclusion criteria, 12 examined COVID-19-related policy impacts on SNAP only, 21 examined impacts on WIC only, and 4 examined impacts on SNAP and WIC. Overall, 18 (48.6%) studies used qualitative approaches, 16 (43.2%) used quantitative approaches, and 3 (8.1%) used mixed methods. Seventeen policies were examined (8 WIC, 9 SNAP) (Table 1). Nine studies (24.3%) used administrative data, 11 used surveys (29.7%), 3 (8.1%) used open-ended survey responses, 15 (40.5%) used interviews, and 5 (13.5%) used focus groups. Five SNAP studies examined the impact of COVID-19-related policies on access, 4 on enrollment/retention, 4 on benefit utilization, and 4 on perceptions; and 8 WIC studies examined the impact of COVID-19-related policies on access, 4 on enrollment/retention, 9 on benefit utilization, and 12 on perceptions. Overall characteristics of studies are in Table 2.

Studies had a mean critical appraisal score of 0.80 (strong), with scores ranging from 0.47 to 1.00. SNAP studies had a mean critical appraisal score of 0.76 (moderate), scores ranging from 0.47 to 1.00. WIC studies had a mean critical appraisal score of 0.80 (strong), scores ranging from 0.50 to 1.00. Critical appraisals are in Table 3 [[22], [23], [24], [25], [26], [27], [28], [29], [30], [31], [32], [33], [34], [35], [36], [37], [38], [39], [40], [41], [42], [43], [44], [45], [46], [47], [48], [49], [50], [51], [52], [53], [54], [55], [56], [57]]. Studies with lower quality appraisal scores were at risk of bias based on this critical appraisal. One cross-sectional study showed potential selection bias due to unclear inclusion criteria and lack of subjects and setting description [32]. Two qualitative studies exhibited risks of bias from unclear congruity between methods and interpretations, as well as the omission of researcher positionality and influence [29,53]. A quasi-experimental study faced confounding bias and attrition bias due to limited adjustments for confounding variables and a 57% follow-up retention rate, with no reported strategies to address missing data [33]. Another study encountered confounding bias as it combined SNAP and pandemic-EBT redemption and did not account for confounders [30].

**TABLE 3:** Summary of studies examining effects of the Supplemental Nutrition Assistance Program (SNAP) and Special Supplemental Nutrition Program on Women, Infants, and Children (WIC) COVID-19 policy flexibilities on Programmatic Outcomes.

Programmatic outcomes of interest	Author and year (QA score)	State	Data source (*n*)	Policies evaluated	Data collection timeframe	Outcome operationalized	Quantitative findings direction/magnitude	Qualitative findings sentiment
SNAP participants (*n* = 13)								
Program access	Barnes and Riel, 2022 [22] (0.80)	NC	IDIs (60)	EAP; NFIW; ECRW	March 2020–August 2020	Reported ease of use and awareness		Mixed
	Barnes, 2023 [23] (0.80)	NC	IDIs (113)	NFIW	June 2020–October 2021	Reported administrative burden		Negative
	Melnick, 2022 [24] (0.70)	47 states	Open-ended survey (146)	EAP	March 2021–May 2021	Pandemic Experiences, SNAP discontinuation		Negative
Program participation	Heflin, 2023 [25] (0.78)	10 states	Caseload data (6020)	IRW	January 2019–May 2021	Caseloads in waiver vs. non-waiver counties	4.8% higher increase	
	Hembre, 2023 [26] (0.83)	Nationwide	Caseload data (4743)	EAP; ECRW; PRRW; IRW; NFIW; EIW; TSRW	January 2014–June 2021	SNAP log of per capita caseloads	19%–22% increase	
	Pukelis, 2023 [27] (1.00)	Nationwide	Caseload data (24)	EAP; ECRW; PRRW; EIW; TSRW	January 2019–November 2022	Effects on enrollment	Only EAP led to increase (3.3–5.7 /1000 HH)	
	Vasan, 2021 [28] (0.88)	40 states	Caseload data (1175)	NFIW; TSRW	January 2019– January 2021	SNAP participation in offline vs. online states	No difference	
Benefit utilization	Cardarelli, 2021 [29] (0.60)	KY	FGs (15)	EAP	May 2021–June 2021	Perceived changes in food purchasing		Positive
	Jones, 2021 [30] (0.67)	Not reported	Redemption data (42)	EAP; ECRW; PRRW; IRW; NFIW; EIW; TSRW; QCIW	January 2017–September 2020	Changes in SNAP monthly redemption	86% higher than previous year	
	Leung and Wolfson, 2023 [31] (0.60)	Not reported	Open-ended survey (415)	TFPI	September 2021–February 2022	Perceived effect		Mixed
Benefit utilization; program perceptions	Babb, 2023 [32] (0.47)	IN	Survey (277); FGs/IDIs (103)	EAP; TFPI	2021; June 2022–July 2022	Perceived benefit adequacy and length of use	79% identified benefits as enough	Mixed
Program perceptions	Adams, 2023 [33] (0.56)	Nationwide	Survey (103)	EAP; ECRW; PRRW	May 2020–May 2021	Perceived benefit	76% EAP, 37.9% PRRW and ECRW by 22.3% as beneficial	
	Spence, 2024 [34] (0.71)	VT	Open-ended Surveys (114)	EAP	July 2020–September 2020	Perceived benefit adequacy		Mixed
SNAP staff (*n* = 3)								
Program access	Bresnahan, 2021 [35] (1.00)	43 states	Survey (43)	EAP; ECRW; PRRW; IRW; NFIW; EIW; TSRW; QCIW	December 2020–January 2021	Perceived effects on services	90% identified flexibilities as critical for service delivery	
	Munger, 2023 [36] (0.80)	OR	IDIs (22)	EAP; NFIW; TSRW	February 2021–May 2021	Perceptions on access		Positive
Program perceptions	Headrick, 2022 [37] (0.80)	26 states	FGs (7)	EAP; ECRW; IRW; NFIW	1 April, 2021	Implementation barriers and facilitators		Mixed
	WIC participants (*n* = 24)							

Program access	Au, 2022 [38] (0.78)	CA	IDIs (182)	PPW; RBI; FPS	June 2020–March 2021	Perceived ease of use		Positive
	Barnes, 2023 [18] (0.50)	LA, MN	IDIs (80)	PPW; RBI; EI; TPC	July 2019–August 2021	Perceived burden and benefit		Negative
	Halverson and Karpyn, 2022 [39] (1.00)	DE	IDIs (51)	PPW; RBI; FPS	March 2022–June 2022	Perceptions on access		Positive
	McElrone, 2020 [40] (1.00)	TN	IDIs (24)	PPW; RBI; FPS	April 2020–May 2020	Early pandemic experiences		Mixed
	Melnick, 2022 [24] (0.70)	47 states	Open-ended survey (149)	PPW	March 2021–May 2021	Pandemic experiences, WIC discontinuation		Negative
	Ritchie, 2021 [41] (1.00)	12 states	Survey (26,642)	PPW; RBI; FPS	March 2021–April 2021	Perceived ease of use	92% were comfortable with remote appointments	
Program access; program participation	Morris, 2021 [42] (0.71)	WA	IDIs (40); Caseload data (72,010)	PPW; RBI; SDW	March 2020–April 2020; December 2019–December 2020	Perceived advantages; changes in participation	11% increase in program participation	Positive
Program participation	Anderson and Whaley, 2023 [43] (1.00)	CA	Surveys (3540)	PPW	July 2020–December 2020	Odds of child recertification	Interactive texting OR: 1.27	
	Vasan, 2021 [28] (0.88)	40 states	Caseload data (1175)	PPW; RBI	January 2019– January 2021	Changes in participation in offline vs. online states	9% decrease in participation	
	Whaley and Anderson, 2021 [44] (0.75)	CA	Caseload data (151)	PPW; RBI; SDW	February 2020–June 2020	Incidence rate ratios of daily certification	24% increase in certification; 27% in recertification	
Benefit utilization	Barnes and Riel, 2022 [22] (0.80)	NC	IDIs (60)	FPS	March 2020–August 2020	Perceived benefit and awareness		Negative
	Chaney, 2024 [45] (0.83)	CA	Redemption data and survey (1463)	CVBI	June 2020–June 2022	Amount and diversity of CVB redemption before (T1), during optional increase (T2), and mandatory increase (T3)	Total FV diversity score increases from T2 vs. T1: 7.82, and T3 vs. T1:6.02.	
	Comi, 2021 [46] (0.80)	NY, NH, NJ, KS	IDIs (4)	FPS	2020–2021	Pandemic experiences		Negative
Benefit utilization; program perceptions	Duffy, 2022 [47] (1.00)	NC	FGs (10)	CVBI	February 2022–March 2022	Perceptions and awareness; redemption		Positive
	Gago, 2022 [48] (1.00)	MA	Surveys (321)	CVBI	February 2022–March 2022	Perceptions of CVB changes and purchasing patterns changes	71% and 55% increased amount and quality of FV purchases; 37.1% more satisfied post-increase	
	Halverson and Karpyn, 2022 [49] (1.00)	DE	IDIs (51)	CVBI	March 2022–June 2022	Satisfaction; benefit redemption		Positive
	Martinez, 2021 [50] (0.80)	CA	IDIs (30)	CVBI	October 2021–December 2021	Satisfaction and purchasing patterns		Positive
	Nitto, 2024 [51] (0.88)	Nationwide	Redemption data (810); interviews (76)	CVBI	May 2020–September 2022	CVB redemption dollar amount and rates; Satisfaction	No differences in redemption rates	Positive
	Whaley, 2023 [52] (0.82)	CA	Surveys (1770)	CVBI	May 2021, September 2021, May 2022	CVB redemption and perceptions of sufficiency before (T1), during optional increase (T2), and mandatory increase (T3)	Perception of sufficiency increased over time (T1: 13.6%; T2: 47.6%; T3: 27.2%)	
Program perceptions	Barnes and Petry, 2021 [53] (0.70)	NC	IDIs (44)	PPW; RBI	March 2020–August 2020	Experiences with remote appointments		Positive
	Barnes, 2023 [23] (0.80)	NC	IDIs (113)	RBI; RBI	June 2020–October 2021	Perceived burden		Positive
	Ritchie, 2022 [54] (0.86)	5 states	Surveys (10,039)	CVBI	October 2020	Satisfaction with $35/mo/participant increase	65% reported high satisfaction	
	Soto Díaz, 2024 [55] (1.00)	NC	IDIs (18)	PPW; RBI; CVBI	August 2022	Experiences and satisfaction		Positive
	Ventura, 2022 [56] (0.75)	CA	Surveys (185)	PPW; RBI	November 2020–February 2021	Satisfaction	84% rated quality of the same or better quality	
WIC staff (*n* = 3)								
Access	Au, 2022 [38] (0.78)	CA	IDIs (22)	PPW; RBI; FPS; SDW	June 2020–March 2021	Perceived advantages		Positive
	Morris, 2021 [42] (0.71)	WA	FGs (52)	PPW; RBI; SDW	March 2020–April 2020	Perceived advantages		Positive
Access; program perceptions	Wrobleska, 2023 [57] (0.71)	Nationwide	Survey (1922)	PPW; RBI; EI; VAF; FPS	March 2021–April 2021	Perceived importance	94.3% state and 87.5% local agency staff reported as extremely important	

Abbreviations: CVBI, WIC Cash-Value Voucher/Benefit Increase; EAP, SNAP Policies: SNAP Emergency Allotments; ECRW, SNAP Extended Certification Periods and adjust Periodic Reports Waiver; EI, WIC Extended Issuance; EIW, SNAP Expedited Interview Waiver; FG: focus group; FPS, food package substitution; HH: household; IDI, in-depth interview; IRW, SNAP Interview Waiver for Initial/Recertification Interview; NFIW, SNAP Interview Waiver to not offer Face-to-Face interview; PPW: WIC policies, WIC Policies: Physical presence waiver; PRRW, SNAP Periodic Reporting Procedures Waiver to Recertify Households; QA: quality appraisal; QCIW, SNAP waiver to conduct quality control interviews in person; RBI, remote benefit issuance waiver; SDW, separation of duties waiver; TFPI, SNAP Thrifty Food Plan Universal Benefit Increase; TPC, WIC Transaction without Presences of a Cashier; TSRW, SNAP Adapted Telephonic Signature Requirements Waiver; VPF, WIC Vendor Preauthorization Flexibilities.

### Results by outcomes

#### Program access

##### SNAP program access

Four studies, 2 in-depth interviews with participants in North Carolina [22,23], 1 with staff [36], and 2 nationwide surveys [24,35], examined how COVID-19 policy changes affected SNAP participants’ program access. One found that most participants were unaware of policies reducing administrative burdens (e.g., face-to-face interview waiver, extended certification periods) [22]. Barriers to participation included technological challenges during recertification [24], perceived insufficient benefit for the effort required [24], and challenges communicating changes in household circumstances to staff [22], among usual challenges exacerbated by the pandemic (e.g., transportation) [23,36]. SNAP staff found policies enabling remote services such as telephonic signatures, waiver of interviews, and certification periods as critical for program accessibility during pandemic onset, but reported challenges to implementation [35]. For example, agencies struggled to retain personnel and monthly approvals for emergency allotments created increased administrative burden and delayed benefit issuance [35].

##### WIC program access

Eight studies explored impact of WIC policy flexibilities on access, with 5 focused on participant perspectives, 1 only on staff, and 2 on both. Two early studies noted limited awareness of policy flexibilities among participants and vendors, with varying levels of awareness depending on the waiver (e.g., greater awareness about physical presence compared with others) [18,40]. Initial service disruptions during the transition to remote services led to long-term access issues, because some participants did not return to the clinics after the transition was complete [18]. However, most studies found that remote services greatly improved convenience, reducing challenges like childcare, transportation, scheduling, and costs, while also helping participants feel safer during the pandemic[18,53,42,41].

State and local agencies highlighted the importance of waivers for physical presence, remote issuance, vendor preauthorization, minimum stocking, routine monitoring, and food substitutions in maintaining quality services, promoting social distancing during pandemic, and improving program accessibility [42,57,58]. WIC staff viewed the shift to remote services positively, noting increased convenience for participants, reduced barriers like transportation and childcare, and improved communication, leading to more in-depth conversations and greater flexibility in meeting participant needs [42,58].

#### Enrollment and retention

##### SNAP enrollment and retention

Four studies evaluated SNAP policy flexibilities on enrollment and retention using caseload data, generally finding increases in participation linked with policy changes [[25], [26], [27], [28]]. Three used state-level data—one found a 19%–22% caseload increase when all flexibilities were adopted, although specific policies had no independent effect [26]. Another study linked emergency allotments to caseload increases, with reductions when the policy lapsed [27]. The third compared participation between states where benefits are loaded at the clinic (offline states) and states where EBT benefits are loaded remotely (online states), but found null results [28]. One study focused on county-level data, showing that counties implementing flexibilities saw a 4.8% higher caseload increase compared with those that did not [25].

##### WIC enrollment and retention

Four studies examined how policy flexibilities affected WIC enrollment and recertification using caseload data or surveys [42,28,43,44]. Two were in California [43,44], 1 in Washington [42], and 1 across 40 states (excluding 10 states in transition to EBT from paper vouchers) [28]. One California study found that the transition to remote services led to a 24% increase in certifications and a 27% increase in recertification, with most racial and ethnic subgroups seeing increases, except Spanish-speaking Hispanic children [44]. The Washington study reported overall participation growth, in all racial and ethnic subgroups, except Alaskan Native children [42]. The other study found that offline states (that is, where participants must visit the clinic in-person to load benefits to their EBT card) experienced a 9% drop in participation after shifting to remote services, compared with online states [28].

#### Benefit utilization

##### SNAP benefit utilization

Four studies examined changes in SNAP benefit utilization related to emergency allotments and Thrifty Food Plan (TFP) increases. One study found an 86.4% increase in SNAP benefit redemption compared with the previous year pre-pandemic [30]. This study combined pandemic-EBT redemptions with SNAP redemptions making it difficult to attribute increases to specific waivers. Two qualitative studies found that increased benefits helped participants purchase more nutritious foods and offset nonfood expenditures [29,31], although rising food prices limited the policies’ impact [31]. For example, at a time when emergency allotments and TFP increases were enacted, participants in Indiana indicated benefits were exhausted by the 13th day of the benefit cycle [32].

##### WIC benefit utilization

Nine studies in California [45,50,52], Delaware [49], Massachusetts [48], North Carolina [22,47], and 2 multistate projects [46,51] examined changes in benefit usage due to increases in the cash-value benefit (CVB) and food package substitutions. Most found that increases led to higher redemption rates with participants buying more, higher quality, and varied produce [[47], [48], [49], [50], [51], [52], ]]. However, studies found that participants still experienced barriers to CVB benefit utilization including lack of physical access to WIC-authorized vendors and difficulty identifying WIC-approved products among urban and rural participants [47,51]. Studies on expanded food package substitutions highlighted challenges participants encountered in redeeming benefits. Early pandemic studies found that WIC vendor staff were often unaware of flexibilities, preventing some participants from benefiting from the waiver [23,46]. Additionally, vendor staff had to verify out of stock items, creating conflicts and further barriers [46]. Some participants felt uncomfortable reporting these challenges to WIC.

#### Program perceptions

##### SNAP program perceptions

One study found most participants identified the TFP increases (79.6%), the streamlined certification process (37.9%), and extended certification (22.3%) as beneficial [33]. Another study identified that at the time when emergency allotments and TFP increases were enacted participants in Indiana still perceived the amount as insufficient [32].

Two studies, 1 multistate and 1 Oregon-based, examined staff perceptions. Although staff broadly identified the flexibilities as important for participants, staff noted challenges with remote services and the need for new technology but cited peer learning networks, partnerships, extra funding, and good communication as helpful in policy implementation [37]. Another study with social services staff found that policy flexibilities helped older adults enroll and afford more nutritious food, although uncertainty about the policy end date caused stress [36].

##### WIC program perceptions

Twelve studies explored WIC participants views on policy flexibilities enabling remote services (*n =* 4, e.g., physical presence waiver, remove benefit issuance) [18,53,57,56], expanding food package substitutions (*n =* 1) [57], and increasing the CVB (*n =* 9) [45,[47], [48], [49], [50], [51], [52],^54^,^55^]. Remote appointments generally led to positive interactions with staff [18,53,42,41]. However, some participants missed anthropometric and hematologic measurement tracking, felt interactions were rushed, and missed in-person connections [23,53,42]. Preference for in-person and remote visits were evenly split and various studies suggested the need for hybrid models [38,42,56]. Phone calls and texting were favored as helpful ways to receive information [38].

Participants were satisfied with the first CVB increase, noting it significantly improved the WIC food package [52,50,47,54,55]. However, satisfaction and perceived sufficiency declined when the amount changed from $35 per participant to variable amounts depending on participant category (e.g., $24 per child participant, $40 per pregnant participant) [52,47].

Staff viewed certain waivers as critical in facilitating services, although some policies were deemed less important in facilitating quality services (e.g., vendor routine monitoring, compliance investigations) [57]. For example, the separation of duties flexibility led to improved customer service and operational efficiency but may have reduced quality assurance [58]. Moreover, issues were identified with extended certification periods as creating confusion among participants [58], and food package substitution waivers confusing to participants and vendors [57,38]. WIC agency directors favored continuing all waivers and changes post-pandemic, particularly remote benefit issuance [42,58].

## Discussion

In this systematic review we examined the impact of COVID-19-related policy flexibilities on program access, enrollment, retention, benefit utilization, and perceptions of SNAP and WIC. Overwhelmingly, results suggest that COVID-19-related policy flexibilities had a positive impact on SNAP and WIC participants’ enrollment, retention, benefit utilization, and perceptions. However, some participant challenges were identified, such as confusion or lack of awareness around changes, technical challenges with program portals during enrollment, and challenges using benefits at the store. For staff issues in participant communication, postal delays with EBT cards, and issuance errors going unnoticed by participants were identified. These challenges should be addressed if policy flexibilities are permanently enacted.

Among studies that report staff perceptions, feedback was predominantly positive with most staff wanting to continue SNAP and WIC flexibilities to reduce administrative burden for themselves and participants. Challenges to address inadequate staffing paired with higher staff turnover and unintended consequences associated with implementing some policies (e.g., backlog from the increased recertification period, increasing workload, and monthly approvals for emergency allotments creating additional logistical barriers).

The primary motivation for this study is identifying ways to address the coverage gaps for SNAP- and WIC-eligible individuals. In recent estimates, 78% of those eligible for SNAP participate [16], and ∼51% of those eligible for WIC participate [17]. COVID-19-related policy flexibilities provide examples of programmatic improvements that may increase enrollment and retention and bridge coverage gaps. A comprehensive examination of the flexibilities’ impact and reach is formative work that aligns with the first pillar of the White House National Strategy on Hunger and Nutrition: “improving food access and affordability” [59]. The reviewed studies indicate opportunities to build upon strategies implemented during the pandemic to improve program and food access for eligible households. The identified lessons learned could be considered by policymakers and program providers as they contemplate a path forward. Although for many individuals COVID-19 may feel like a distant past, in 2024, higher food costs and reports of food insecurity are increasing significantly across the United States [60]. Participation in federal food and nutrition assistance programs has been shown to support household food security and lifelong nutrition [8]. Implementing policies that increase access and reduce barriers to participation and retention could have a direct impact on household food security and a downstream impact on chronic conditions that stem from food insecurity.

### Strengths

Strengths include the reproducibility of the work and findings as the research question and protocol are pre-registered on PROSPERO (CRD42023493302). All procedures are outlined in the protocol, including Boolean search codes and detailed explanations of data extraction and synthesis to encourage replication and open science. Another strength is the opportunity to synthesize literature across databases and platforms, including gray literature and dissertations. Given that WIC and SNAP are federal programs, strong analytic evaluations are often conducted by the federal government, their contractors, and organizations that support these programs. Results from those studies may not be captured through the typical peer-review process but are valuable for understanding participant and staff perceptions and outcomes. A final strength is the extensive data extraction and quality appraisal process used to analyze and evaluate findings. Two independent reviewers completed extraction for each article and 2 additional reviewers conducted quality appraisals. The JBI critical appraisal tools were used by reviewers to assess trustworthiness, relevance, and results of published papers before inclusion [21]. This allowed the reviewers and authors to ensure that regardless of article source, studies included were of high quality and relevant to the research question.

### Limitations

One limitation is that implementation of policies is variable and contextual, based on each states’ interpretation, capacity to implement, and political and public opinions or attitudes toward assistance programs. Many studies explore counts of client records, but there are contextual factors, some listed in the prior sentence, that shape access, enrollment, and retention. Although it is impossible to control for all factors, many studies have statistically controlled for some and the qualitative data on staff perceptions shed light on practical limitations to implementation. In addition, a handful of studies included in the review showed potential risk of bias, including selection bias, confounding bias, and issues with methodological congruity with the qualitative interpretation. We accounted for these limitations by carefully considering the strengths and weaknesses of each study when interpreting the overall findings.

### Future directions

One important research question is whether increased participation and retention stemming from policy flexibilities improves participant and household nutritional status, quality of foods consumed, and downstream health outcomes. In April 2024, the USDA announced permanent changes to the WIC food package expanding pandemic-related increases in CVB for fruits and vegetables. Preliminary evidence from our review suggests that increased benefits for fruits and vegetables may have improved participant satisfaction, benefit redemption, and consumption and recent evidence from others suggests that the policy change may improve household food security [61]. Future research should continue to assess how these changes impact relevant maternal-child health outcomes.

Another important next step could be calculating the cost effectiveness of temporary WIC and SNAP flexibilities. In December 2023, the White House announced an initiative to “Advance the Frontiers of Benefit-Cost Analysis and Strengthen Government Decision Making” [62]. Understanding which policy flexibilities are most cost effective at improving broader social and health effects can guide administrative planning around permanent policy flexibilities and population health. For example, Kenney et al. [63] reported that improving the WIC food package in 2009 prevented ∼62,000 cases of childhood obesity among children from households with lower income. Similar analyses could be done assessing policy flexibilities’ impact on food insecurity, days of missed work, obesity, type 2 diabetes, and other diet-related diseases. Lastly, some policies have more support than others due to being studied and published more often. We found policies that directly impacted services for participants, such as those that enabled remote services, reduced participant administrative burden, and increased benefit amounts, appeared more often than policies intended to enhance program integrity or reduce workload for staff or authorized vendors. Future research should attempt to isolate policies that were enacted, but less-frequently evaluated, such as separation of duties and WIC vendor authorization flexibility.

### Conclusion

COVID-19-related policy flexibilities illustrate valuable programmatic improvements that show promise in enhancing program access and participation, aligning with national strategies to improve food access and affordability, and should be considered by policymakers to address ongoing food and nutrition insecurity challenges. Policy flexibilities, such as increases to SNAP benefit and simplified recertification processes, were found to significantly enhance program participation and benefit redemption, despite some implementation challenges. Similarly, WIC policy flexibilities, including remote service provisions and increased CVB, improved program accessibility, increased access to fruits and vegetables, and participant satisfaction. The quick response of the COVID-19 public health emergency to ensure SNAP and WIC services could be delivered safely to meet the needs of eligible households has provided important lessons for the ongoing betterment of the programs. Future efforts should continue to evaluate long-term effects of flexibilities on nutrition and health outcomes, cost-effectiveness, and explore less studied policies to inform permanent program adjustments.

## Author contributions

The authors’ responsibilities were as follows – MC-B, GH: designed the study protocol and trained research assistants; AK, SS: conducted title, abstract, and full-text screening, with discrepancies resolved by content experts MC-B, GH, KH-H; all team members: participated in data extraction; MC-B, GH, KH-H, JH, MAL: conducted quality appraisals; MC-B, JH: conducted data synthesis; MC-B, GH, MAL, JH, KH-H: wrote the paper; and all authors: revised, read, and approved the final manuscript.

## Funding

This paper was supported by Healthy Eating Research, a national program of the Robert Wood Johnson Foundation.

## Conflicts of interest

The authors report no conflicts of interest.

## References

[1] SNAP Data Tables. Food and nutrition service [Internet]. [cited 7 May, 2024]. Available from: https://www.fns.usda.gov/pd/supplemental-nutrition-assistance-program-snap. 2023

[2] WIC Data Tables. Food and nutrition service [Internet]. [cited 7 May, 2024]. Available from: https://www.fns.usda.gov/pd/wic-program. 2023.

[3] SNAP Eligibility. Food and nutrition service [Internet]. [cited 7 May, 2024]. Available from: https://www.fns.usda.gov/snap/recipient/eligibility. 2024.

[4] WIC Eligibility Requirements. Food and nutrition service [Internet]. [cited 7 May, 2024]. Available from: https://www.fns.usda.gov/wic/wic-eligibility-requirements. 2024.

[5] M. Nord, A.M. Golla, Does SNAP decrease food insecurity? Untangling the self-selection effect [Internet]. USDA Economic Research Service; Report No.: 85 [1 October, 2009; 7 May, 2024]. Available from: https://www.ers.usda.gov/webdocs/publications/46295/10977_err85_1_.pdf?v=0.

[6] Ratcliffe C., McKernan S.-M., Zhang S. (2011). How much does the Supplemental Nutrition Assistance Program reduce food insecurity. Am. J. Agric. Econ..

[7] Arteaga I., Heflin C., Gable S. (2016). The impact of aging out of WIC on food security in households with children, Child Youth Serv. Rev.

[8] Insolera N., Cohen A., Wolfson J.A. (2022). SNAP and WIC participation during childhood and food security in adulthood, 1984–2019. Am. J. Public Health..

[9] Whiteman E.D., Chrisinger B.W., Hillier A. (2018). Diet quality over the monthly Supplemental Nutrition Assistance Program cycle. Am. J. Prev. Med..

[10] Lora K.R., Hodges L., Ryan C., Ver Ploeg M., Guthrie J. (2023). Factors that influence children’s exits from the Special Supplemental Nutrition Program for Women, Infants, and Children: a systematic review. Nutrients.

[11] SNAP Food Security In-Depth Interview Study. Food and nutrition service [Internet]. [cited 7 May, 2024]. Available from: https://www.fns.usda.gov/snap/food-security-depth-interview-study.

[12] Gaines-Turner T., Simmons J.C., Chilton M. (2019). Recommendations from SNAP participants to improve wages and end stigma. Am. J. Public Health..

[13] Zhang Q., Alsuliman M.A., Wright M., Wang Y., Cheng X. (2020). Fruit and vegetable purchases and consumption among WIC participants after the 2009 WIC food package revision: a systematic review. Adv. Nutr..

[14] M. Nord, M. Prell, Food security improved following the 2009 ARRA increase in SNAP benefits [Internet]. [cited 7 May, 2024]. Available from: http://www.ers.usda.gov/publications/pub-details/?pubid=44839. 2011.

[15] How administrative burdens can harm health. Health affairs brief [Internet]. [cited 7 May, 2024]. Available from: https://www.healthaffairs.org/do/10.1377/hpb20200904.405159/full/

[16] Reaching Those in Need: Estimates of State Supplemental Nutrition Assistance Program Participation Rates in 2020 [Internet]. Mathematica. [cited 7 May, 2024]. Available from: https://www.mathematica.org/publications/estimates-of-state-supplemental-nutrition-assistance-program-participation-rates-in-2020. 2023.

[17] National and State Level Estimates of WIC Eligibility and Program Reach in 2021 Food and Nutrition Service [Internet]. [cited 7 May, 2024]. Available from: https://www.fns.usda.gov/research/wic/eligibility-and-program-reach-estimates-2021. 2024.

[18] Barnes C., Halpern-Meekin S., Hoiting J. (2023). “I Used to Get WIC. But Then I Stopped”: how WIC participants perceive the value and burdens of maintaining benefits. RSF..

[19] Rep. Lowey NM [D-N-17. H.R.6201 - 116th Congress (2019-2020): Families First Coronavirus Response Act Internet [cited 7 May, 2024]. Available from: 2020. https://www.congress.gov/bill/116th-congress/house-bill/6201. 2020.

[20] States Are Using Much-Needed Temporary Flexibility in SNAP to Respond to COVID-19 Challenges Center on Budget and Policy Priorities Internet [cited 7 May, 2024]. Available from: 2020. https://www.cbpp.org/research/food-assistance/most-states-are-using-new-flexibility-in-snap-to-respond-to-covid-19. 2023.

[21] Barker T.H., Stone J.C., Sears K., Klugar M., Leonardi-Bee J., Tufanaru C. (2023). Revising the JBI quantitative critical appraisal tools to improve their applicability: an overview of methods and the development process. JBI Evid. Synth..

[22] Barnes C., Riel V. (2022). ‘I don’t know nothing about that’: how “learning costs” undermine COVID-related efforts to make SNAP and WIC more accessible. Adm. Soc..

[23] Barnes C. (2023). “I can’t get ahold of them”: perceptions of administrative burden and administrative exclusion across SNAP, WIC, and Medicaid during the COVID-19 pandemic. Ann. Am. Acad. Pol. Soc. Sci..

[24] Melnick E.M., Ganderats-Fuentes M., Ohri-Vachaspati P. (2022). Federal food assistance program participation during the COVID-19 pandemic: participant perspectives and reasons for discontinuing. Nutrients.

[25] Heflin C., Fannin W.C., Lopoo L. (2023). Local control, discretion, and administrative burden: SNAP interview waivers and caseloads during the COVID-19 pandemic. Am. Rev. Public Adm.

[26] Hembre E. (2023). Examining SNAP and TANF caseload trends, responsiveness, and policies during the COVID-19 pandemic. Contemp. Econ. Policy..

[27] Pukelis K. (2023).

[28] Vasan A., Kenyon C.C., Roberto C.A., Fiks A.G., Venkataramani A.S. (2021). Association of remote vs in-person benefit delivery with WIC participation during the COVID-19 pandemic. JAMA.

[29] Cardarelli K.M., DeWitt E., Gillespie R., Graham R.H., Norman-Burgdolf H., Mullins J.T. (2021). Policy implications of the COVID-19 pandemic on food insecurity in rural America: evidence from Appalachia. Int. J. Environ. Res. Public Health..

[30] Jones J.W. (2021). IDEAS Work Pap Ser RePEc.

[31] Leung C.W., Wolfson J.A. (2023). The impact of the 2021 Thrifty Food Plan benefit re-evaluation on SNAP participants' short-term food security and health outcomes. Front Public Health.

[32] Babb A.M., Suttles S.A., Daellenbach I., DuPilka J.H., Knudsen D.C. (2023). Adequacy of SNAP benefits for Indiana households. Am. J. Public Health..

[33] Adams E.L., Caccavale L.J., Smith D.I., Bean M.K. (2023). Food insecurity, federal nutrition support, and parent feeding practices during COVID-19: a 1-year follow-up study. Public Health Rep.

[34] Spence E.H., Niles M.T., Bertmann F., Belarmino E.H. (2024).

[35] Bresnahan C., Ellison C., Green C., Headrick G., Ji Yeun Lee C., Lyons M. (2021). https://files.constantcontact.com/391325ca001/43b432bd-bdde-4525-8e63-a1b0293de236.pdf.

[36] Munger A.L., Speirs K.E., Grutzmacher S.K., Edwards M. (2023). Social service providers’ perceptions of older adults’ food access during COVID-19. J. Aging Soc. Policy..

[37] Headrick G., Ellison C., Bresnahan C., Green C., Lyons M., Moran A. (2022). State implementation of SNAP waivers and flexibilities during the COVID-19 pandemic: perspectives from state agency leaders. J. Nutr. Educ. Behav..

[38] Au L.E., Whaley S.E., Hecht C.A., Tsai M.M., Anderson C.E., Chaney A.M., Vital N., Martinez C.E., Ritchie L.D. (2022). California WIC participants’ and local agency directors’ experiences during the coronavirus disease 2019 pandemic: a qualitative examination. J. Acad. Nutr. Diet..

[39] Halverson M.M., Karpyn A. (2023). Pandemic-era WIC participation in wilmington, delaware: participants’ experiences and challenges. Nutrients.

[40] McElrone M., Zimmer M.C., Anderson Steeves E.T. (2021). A qualitative exploration of predominantly white non-Hispanic Tennessee WIC participants’ food retail and WIC clinic experiences during COVID-19. J. Acad. Nutr. Diet..

[41] Ritchie L.D., Lee D., Sallack L., Chauvenet C., Machell G., Kim L. (2021). Multi-state WIC participant satisfaction survey: learning from program adaptations during COVID. https://thewichub.org/multi-state-wic-participant-satisfaction-survey-learning-from-program-adaptations-during-covid/.

[42] Morris E.J., Quinn E.L., Rose C.M., Spiker M., O’Leary J., Otten J.J. (2022). Insights from Washington State’s COVID-19 response: a mixed-methods evaluation of WIC remote services and expanded food options using the RE-AIM framework. J. Acad. Nutr. Diet..

[43] Anderson C.E., Whaley S.E. (2023). Use of interactive texting is associated with higher odds of continued WIC participation during the COVID-19 pandemic. J. Acad. Nutr. Diet..

[44] Whaley S.E., Anderson C.E. (2021). The importance of federal waivers and technology in ensuring access to WIC during COVID-19. Am. J. Public Health..

[45] Chaney A.M., Anderson C.E., Arnold C.D., Whaley S.E., Ritchie L.D., Pundi G.R. (2024). Evaluating the association of the increase in the WIC cash value benefit on the diversity of myplate fruits and vegetables redeemed and consumed by children in low-income households. Curr. Dev. Nutr..

[46] Comi D. (2021). http://proxygw.wrlc.org/login?url=https://www.proquest.com/dissertations-theses/locating-when-access-through-genre-infrastructure/docview/2596937426/se-2?accountid=11243.

[47] Duffy E.W., Vest D.A., Davis C.R., Hall M.G., De Marco M., Ng S.W. (2022). “I Think That’s the Most Beneficial Change That WIC Has Made in a Really Long Time”: perceptions and awareness of an increase in the WIC cash value benefit. Int. J. Environ. Res. Public Health..

[48] Gago C., Colchamiro R., May K., Rimm E.B., Kenney E.L. (2022). Caregivers’ perceived impact of WIC’s temporary Cash-Value Benefit (CVB) increases on fruit and vegetable purchasing, consumption, and access in Massachusetts. Nutrients.

[49] Halverson M.M., Karpyn A. (2022). WIC participants’ perceptions of the cash-value benefit increase during the COVID-19 pandemic. Nutrients.

[50] Martinez C.E., Ritchie L.D., Lee D.L., Tsai M.M., Anderson C.E., Whaley S.E. (2022). California WIC participants report favorable impacts of the COVID-related increase to the WIC cash value benefit. Int. J. Environ. Res. Public Health..

[51] Nitto A.M., Crespo-Bellido M., Yenerall J., Anderson Steeves E.T., Kersten S.K., Vest D. (2024). Mixed methods evaluation of the COVID-19 changes to the WIC cash-value benefit for fruits and vegetables. Front Public Health.

[52] Whaley S.E., Anderson C.E., Tsai M.M., Yepez C.E., Ritchie L.D., Au L.E. (2023). Increased WIC benefits for fruits and vegetables increases food security and satisfaction among California households with young children. J. Acad. Nutr. Diet..

[53] Barnes C., Petry S. (2021). “It Was Actually Pretty Easy”: COVID-19 compliance cost reductions in the WIC program, Public Adm. Rev..

[54] Ritchie L.D., Lee D., Felix C., Sallack L., Chauvenet C., Machell G. (2022). Multi-state WIC participant satisfaction survey: cash value benefit increase during COVID. https://thewichub.org/multi-state-wic-participant-satisfaction-survey-cash-value-benefit-increasing-during-covid/.

[55] Soto Díaz C.R., Taillie L.S., Higgins I.C.A., Richter A.P.C., Davis C.R., De Marco M. (2024). A qualitative exploration of Spanish-speaking Latina women’s experiences participating in WIC before and during the COVID-19 pandemic. J. Acad. Nutr. Diet..

[56] Ventura A.K., Martinez C.E., Whaley S.E. (2022). WIC participants’ perceptions of COVID-19-related changes to WIC recertification and service delivery. J. Community Health..

[57] Wroblewska K., Steigelman C., Hansen D. (2023). The use and impact of federal waivers during the COVID-19 pandemic: summary findings from surveys of WIC state and local agencies | food and nutrition service. https://www.fns.usda.gov/research/wic/impact-federal-waivers-pandemic.

[58] Au L., Ritchie L., Vital N., Tsai M., Anderson C., Meza M. (2021). WIC is critical during the COVID-19 pandemic: lessons learned from Los Angeles county participants, Curr. Dev. Nutr..

[59] House T.W. (2022). EXECUTIVE SUMMARY: Biden-Harris administration national strategy on hunger, nutrition, and health. The White House.

[60] USDA ERS. Food price outlook [Internet]. [cited 14 May, 2024]. Available from: https://www.ers.usda.gov/data-products/food-price-outlook/.

[61] Tsai M.M., Anderson C.E., Whaley S.E., Yepez C.E., Ritchie L.D., Au L.E. (2024). Associations of increased WIC benefits for fruits and vegetables with food security and satisfaction by race and ethnicity. Prev. Chronic. Dis..

[62] FACT SHEET (2023). https://www.whitehouse.gov/omb/briefing-room/2023/12/14/fact-sheet-biden-harris-administration-announces-new-initiative-to-advance-the-frontiers-of-benefit-cost-analysis-and-strengthen-government-decision-making/.

[63] Kenney E.L., Lee M.M., Barrett J.L., Ward Z.J., Long M.W., Cradock A.L. (2024). Cost-effectiveness of improved WIC food package for preventing childhood obesity. Pediatrics.

