# Supplementary material for: A Systematic Review: The Impact of COVID-19 Policy Flexibilities on SNAP and WIC Programmatic Outcomes
Source: Adv Nutr. 2024 Dec 20;16(2):100361. doi: 10.1016/j.advnut.2024.100361 (PMC11773222; doi:10.1016/j.advnut.2024.100361)
Supplement: Multimedia component 1 [file mmc1.docx]

Supplemental Table 1. Example Boolean Search Strings used to gather Included Literature

| Program | Example Boolean Search String |
| --- | --- |
| Supplemental Nutrition Assistance Program (SNAP) | (“SNAP” OR “Supplemental Nutrition Assistance Program” OR “Electronic Benefit Transfer” OR “EBT”) AND (“COVID-19” OR “Pandemic” OR “Corona”) AND (“Change*” OR “Changed” OR “Waive*” OR “Flexibilit*”) AND (“Redemp*” OR “Access*” OR “Recert*” OR “Use*” Or “Using” OR “Appl*” OR “Application” OR “Satisfaction” OR “Enroll*” OR “Issu*” OR “Implement*”) |
| Special Supplemental Nutrition Program for Women, Infants, and Children (WIC) | ("Special program for women, infants and children" OR "WIC" OR "EBT" OR "Electronic benefit transfer" OR "FMNP" ) AND ( "COVID-19" OR "corona*" OR "pandemic" ) AND ( "Changes" OR "Waivers" OR "Flexibilities" OR "Remote" OR "Cash-value benefit" OR "Telehealth" OR "Modifications" ) AND ( "Perception" OR "redemp*" OR "Engag*" OR "Satisfaction" OR "Enroll*" OR "Retention" OR "issu*" OR "distribution" ) AND NOT ( "infection" OR "epidemiology" OR "clinical trial" ) |
